# Impact of body fat distribution on neoadjuvant chemotherapy outcomes in advanced breast cancer patients

**DOI:** 10.1002/cam4.571

**Published:** 2015-12-02

**Authors:** Toshiaki Iwase, Takafumi Sangai, Takeshi Nagashima, Masahiro Sakakibara, Junta Sakakibara, Shouko Hayama, Emi Ishigami, Takahito Masuda, Masaru Miyazaki

**Affiliations:** ^1^ Department of General SurgeryChiba Graduate School of Medicine2608677 1‐8‐1 Inohana Chuo‐KuChiba CityChibaJapan

**Keywords:** Body composition, breast neoplasms, menopause, neoadjuvant therapy, survival

## Abstract

Obesity is known to decrease the efficacy of neoadjuvant chemotherapy (NAC) against breast cancer; however, the relationship between actual body composition and NAC outcomes remains unknown. Therefore, we determined the effect of body composition on NAC outcomes. A total of 172 advanced breast cancer patients who underwent surgery after NAC were retrospectively analyzed. Body composition parameters including abdominal circumference (AC), subcutaneous fat area (SFA), visceral fat area (VFA), and skeletal muscle area (SMA) were calculated using computed tomography volume‐analyzing software. VFA/SFA ratio was used to evaluate visceral obesity. The associations of body composition parameters with pathological complete remission (pCR) and survival were analyzed. AC, SFA, and VFA were significantly correlated with body mass index (BMI) (all *P* < 0.05; *r* = 0.82, *r* = 0.71, and *r* = 0.78, respectively). AC, SFA, and VFA increased significantly and SMA decreased significantly after menopause (all *P* < 0.05). VFA/SFA ratio increased significantly after menopause, even though BMI remained unchanged. Body composition parameters were not associated with pCR. Distant disease‐free survival (DDFS) was significantly worse in the high VFA group than in the low VFA group (*P* < 0.05). Furthermore, in the high VFA group, postmenopausal patients had significantly shorter DDFS than premenopausal patients (*P* < 0.05). VFA was independently associated with DDFS in the multivariate analysis (*P* < 0.05). High visceral fat is associated with worse NAC outcomes in breast cancer patients, especially postmenopausal patients. Interventions targeting visceral fat accumulation will likely improve NAC outcomes.

## Background

Obesity not only increases breast cancer risk but also reduces chemotherapy efficacy 1, 2. The pathological complete response (pCR) and disease‐free survival rates after neoadjuvant chemotherapy (NAC) tend to be lower among obese patients 1, 3. Thus, understanding the mechanisms by which obesity negatively affects chemotherapy outcomes is essential in improving the prognosis of obese breast cancer patients. The World Health Organization (WHO) defines obesity as a body mass index (BMI) > 25 kg/m^2^. BMI is a simple and reliable surrogate measure of obesity; however, this index is not a substitute for actual body fat distribution (BFD), as it is based only on weight and height.

Body fat is generally distributed viscerally, subcutaneously, and internally (mostly in the liver), and the BFD pattern differs between individuals 4, 5. Furthermore, the endocrine function of fat cells differs according to the anatomical site. For example, dysfunctional visceral fat cells promote tumor progression by increasing inflammatory cytokines (interleukin‐6 and tumor necrosis factor‐*α*) and endocrine products (aromatase and adiponectin) through activation of the phosphatidylinositol‐3 kinase/Akt pathway 6. On the other hand, excess fat accumulation in the liver (i.e., fatty liver) worsens insulin resistance and promotes tumor cell proliferation and survival by activating the insulin‐like growth factor pathway 7. Given these facts, individual patient BFD is an important consideration in obesity research. Changes in body composition are known to reduce NAC efficacy in the clinical setting. However, to date, only a few studies have investigated the association between BFD and chemotherapeutic efficacy 8, 9.

Therefore, in this study, we aimed to clarify the effect of actual BFD on NAC outcomes in breast cancer patients.

## Materials and Methods

### Inclusion criteria

One hundred and seventy‐two advanced breast cancer patients who underwent surgery after NAC between January 2004 and December 2012 were included in this study. The inclusion criteria were as follows: Eastern Cooperative Oncology Group performance status of 0−1, a clinical T2 classification or higher (tumor size ≥ 20 mm) based on the American Joint Committee on Cancer/Union for International Cancer Control TNM classification 10, and a positive axillary lymph node proven by fine needle aspiration cytology. Core needle biopsy specimens were obtained from all patients before NAC, and were analyzed immunohistochemically for histological type, grade, and subtype. Patients with distant metastasis, identified based on computed tomography (CT) and ^99m^technetium bone scans performed before NAC, were excluded from the study.

The study protocol was approved by our institutional ethics committee and conducted in accordance with the Declaration of Helsinki. Informed consent was obtained from each patient.

### Evaluation of body fat distribution

The WHO classification was used to categorize patients into four weight groups according to BMI: underweight (BMI < 18.5 kg/m^2^), normal (BMI ≥ 18.5 and <25 kg/m^2^), overweight (BMI ≥ 25 and <30 kg/m^2^), and obese (BMI ≥ 30 kg/m^2^). CT scans taken at the time of initial treatment were analyzed for actual BFD using a CT image analyzer (CYNAPSE VINCENT^®^, Fujifilm Co. Ltd., Tokyo, Japan). The subcutaneous fat area (SFA, [cm^2^]) and visceral fat area (VFA [cm^2^]) were calculated using axial cross‐sectional CT images taken at the navel level and used to estimate subcutaneous and visceral fat mass. In addition to SFA and VFA, the degree of muscle mass, a major component of body composition, was analyzed. The lumbar muscle cross‐sectional area (LMCA [m^2^]) was used as a measure of total body skeletal muscle mass and was quantified by CT analysis at the third lumbar vertebrae level (L3) with a Hounsfield unit threshold range −29 to +150 11. The LMCA included the psoas, paraspinal muscles (erector spinae, quadratus lumborum), and abdominal wall muscles (transversus abdominus, external and internal obliques, and rectus abdominus). To adjust the total L3 skeletal muscle mass according to patient stature, the lumbar skeletal muscle index (LSMI) was used. To calculate the LSMI, the LMCA was normalized to the body surface area, which is linearly related to the total body muscle mass (LSMI = total L3 skeletal muscle mass/body surface area [cm^2^/m^2^]) 12. Fat accumulation in the liver was used as a measure of internal fat distribution. The CT liver‐to‐spleen attenuation ratio (liver/spleen [L/S] ratio) was used for evaluation of fatty liver. Fatty liver was defined as an L/S ratio of >1.0 13.

### Neoadjuvant chemotherapy regimen and dosing

The NAC regimen consisted of an anthracycline followed by a taxane in accordance with the results of major clinical trials 14. Patients with human epidermal growth factor receptor 2 (HER2)‐expressing tumors were administered trastuzumab for 1 year 15, 16. The chemotherapy dose was based on the actual weight, regardless of the patient's body composition. Adverse events that occurred during NAC were evaluated according to the National Cancer Institute Common Terminology Criteria for Adverse Events version 4.0 and recorded. The dose intensity and relative dose intensity (RDI) were calculated using electronically recorded data. The dose intensity (mg/m^2^/week) was calculated using the following formula: total dose (mg/m^2^)/duration of administration (weeks). RDI was calculated as follows: (dose intensity/planned dose intensity) × 100.

### Pathological evaluation

All histopathological evaluations were performed by board‐certified pathologists. The definition of pCR has not yet been established, and several definitions based on residual tumor status in the axillary lymph nodes and local tumor site exist 17. In this study, we used the following two definitions for pCR: no residual tumor cells in the breast (ypT0) and noninvasive residuals allowed in the intraductal components (ypTis/mic), regardless of the axillary lymph node status (ypN+) 18, 19.

### Statistical methods

This study was designed as a retrospective patient control study. Pearson's correlation test was used to analyze the correlation between BFD parameters and BMI (*n* = 172). Changes in BFD parameters after menopause were analyzed using Student's *t*‐test (*n* = 172). As previous studies did not identify definitive cut‐off values for VFA and SFA, we instead used a cut‐off value of 100 cm^2^ for both parameters based on the Guideline for Screening Metabolic Disease created by the Japan Society for the Study of Obesity 20. The guideline CT‐based cut‐off values were generated based on morbidity risks in 1193 patients 20. As dual‐energy X‐ray absorptiometry was not performed, CT analysis was also used to measure skeletal muscle mass. Sarcopenia was defined as a skeletal muscle mass of at least two standard deviations lower than that of the normal population 21. Using this definition, this study included only five sarcopenic patients. Therefore, we alternatively used the term “reduced muscle mass,” which was defined as an LSMI less than or equal to the lower quartile for all patients.

Kaplan–Meier survival analysis was performed to evaluate the effect of BFD parameters on survival outcomes (*n* = 172). Distant disease‐free survival (DDFS) was defined as the time from initial NAC treatment to relapse at any distant site. Multivariate analysis by Cox proportional hazards regression model was used to determine the associations of BMI, BFD parameters, and clinicopathological factors with DDFS and overall survival (*n* = 172). Hazard ratios (HRs) and their 95% confidence intervals (CIs) were also calculated. All statistical analyses were performed using SPSS Statistics^®^ version 23 (IBM Co. Ltd., Tokyo, Japan). A *P* value of <0.05 was considered significant, and all statistical tests were two‐sided.

## Results

### Patient characteristics

The average BMI was 22.9 kg/m^2^. According to the WHO classification, 111 (65%), 18 (10%), 35 (20%), and 8 (5%) patients were normal weight, underweight, overweight, and obese, respectively. We determined the distribution of breast cancer subtypes to evaluate the selection bias. The subtypes were as follows: estrogen receptor (ER)‐positive (+)/HER2‐negative (−) in 45% of patients; ER+/HER2+ in 17% of patients; ER‐/HER2+ in 20% of patients; and triple‐negative in 18% of patients (Table 1). This distribution of breast cancer subtypes is similar to that in previous studies 22, 23, suggesting that selection bias was minimal in our study.

**Table 1:** Patient demographics

Variables	No. of patients (*n *=* *172)	(%)
Age, years (median, range)	54 (30–76)	
BMI, kg/m^2^ (mean ± SD)	22.9 ± 3.8	
WHO BMI classification		
Underweight (BMI < 18.5 kg/m^2^)	18	(10)
Normal range (18.5 ≤ BMI < 25 kg/m^2^)	111	(65)
Overweight (25 ≤ BMI < 30 kg/m^2^)	35	(20)
Obese (BMI ≥ 30 kg/m^2^)	8	(5)
Subcutaneous fat area, cm^2^ (mean ± SD)	138.2 ± 65.3	
Visceral fat area, cm^2^ (median, range)	73.0 (5–317)	
V/S ratio, % (median, range)	52 (15–223)	
Lumber skeletal muscle index, cm^2^/cm^3^ (mean ± SD)	57.5 ± 7.5	
Liver/spleen HU ratio (median, range)	1.3 (0.1–2.0)	
Menopausal status		
Premenopausal	74	(43)
Postmenopausal	98	(57)
T		
1	0	(0)
2	119	(69)
3	24	(14)
4	28	(16)
Histological type		
Ductal	140	(81)
Other	32	(19)
Subtype		
ER (+), HER2 (−)	77	(45)
ER (+), HER2 (+)	30	(17)
HER2	34	(20)
Triple‐negative	31	(18)

BMI, body mass index; SD, standard deviation; WHO, World Health Organization; V/S ratio, visceral/subcutaneous fat ratio; HU, Hounsfield unit; ER, estrogen receptor; HER2, human epidermal growth factor receptor 2.

### Body fat distribution

In the BFD analysis, the average SFA was 138.2 cm^2^ and the median VFA was 73.0 cm^2^. To further evaluate BFD, we calculated the VFA/SFA ratio. Although the SFA was much higher than the VFA, the median VFA/SFA ratio was 52%, indicating an almost equal visceral and subcutaneous fat distribution. The median L/S ratio was 1.3, and 18 patients had fatty liver. Table 2 shows the results of the correlation analyses between the BMI and BFD parameters. BMI significantly correlated with the SFA, VFA, VFA/SFA ratio, and L/S ratio (*r* = 0.72, 0.62, 0.27, and −0.45, respectively; all *P* < 0.05). In contrast, muscle mass (LSMI) did not significantly correlate with BMI (*P* = 0.19; Table 2).

**Table 2:** Results of the correlation analyses for BMI and body fat distribution parameters, and univariate analyses for body fat distribution changes after menopause

Variables	Correlation to BMI Pearson's R	*P* b	Premenopausal patients (*n *=* *74)	Postmenopausal patients (*n *=* *98)	*P* a
BMI, kg/m^2^ (mean ± SD)			22.2 ± 3.6	23.4 ± 3.9	0.053
Subcutaneous fat area, m^2^ (mean ± SD)	0.72	<0.05	126.6 ± 67.8	146.8 ± 63.7	<0.05
Visceral fat area, m^2^ (median, range)	0.62	<0.05	45 (5–235)	89 (9–317)	<0.05
V/S ratio, % (median, range)	0.27	<0.05	46 (15–223)	63 (16–184)	<0.05
Lumber skeletal muscle index, cm^2^/cm^3^ (mean ± SD)	−0.1	0.19	61.8 ± 6.9	54.3 ± 6.2	<0.05
Liver/spleen HU ratio (median, range)	−0.45	<0.05	1.3 (0.1–2)	1.3 (1.0–2)	0.78

BMI, body mass index; SD, standard deviation; V/S ratio, visceral fat area/subcutaneous fat area ratio; HU, Hounsfield unit.

a Student's t‐test.

b Pearson's correlation coefficient.

Next, we analyzed the changes in body composition between pre‐ and postmenopausal women. The SFA and VFA increased significantly, whereas the LSMI decreased significantly after menopause (all *P* < 0.05). In addition, the VFA/SFA ratio increased significantly after menopause, indicating that the amount of visceral fat exceeded that of subcutaneous fat (*P* < 0.05). In contrast, the BMI and L/S ratio were not influenced by the menopausal status. These results indicate that menopause causes marked changes in body composition, including reduced muscle mass and increased visceral fat.

### Body fat distribution and pathological complete response

Of the 172 patients, 46 achieved pCR. The pCR rates for patients with ER+/HER2−, ER+/HER2+, ER−/HER2+, and triple‐negative subtypes were 12% (9/77), 30% (9/30), 50% (17/34), and 35% (11/31), respectively. Table 3 shows the results of the univariate analysis of the association of pCR with BMI, BFD parameters, and clinicopathological factors. No significant relationship was observed between pCR and BMI (chi‐squared test; *P* = 0.88). Moreover, the BFD parameters were not associated with pCR. Among the clinicopathological factors, the histological type and subtype were significantly associated with pCR (*P* < 0.05; Table 3).

**Table 3:** Univariate analyses of the associations between pCR and body fat distribution parameters

Variables	No. of non‐pCR patients (*n *=* *126)	%	No. of pCR patients (*n *=* *46)	%	*P*
Age (year, median, range)	55 (30–76)		53 (32–75)		0.63
BMI (kg/m^2^, average, ±SD)	22.8 ± 3.9		23.0 ± 3.7		0.74
WHO BMI classification					
Underweight (BMI < 18.5 kg/m^2^)	14	(11)	4	(9)	0.88
Normal range (18.5 ≤ BMI < 25 kg/m^2^)	81	(64)	30	(65)	
Overweight (25 ≤ BMI < 30 kg/m^2^)	26	(21)	9	(20)	
Obese (BMI ≥ 30 kg/m^2^)	5	(4)	3	(7)	
Subcutaneous fat area (cm^2^, average ± SD)	134 ± 66		148 ± 64		0.22
Visceral fat area (cm^2^, median, range)	73 (5–317)		81 (10–217)		0.53
V/S ratio (%, median, range)	54.0 (15–223)		47.5 (19–127)		0.32
Lumber skeletal muscle index (cm^2^/cm^3^, average ± SD)	57.5 ± 7.6		57.6 ± 7.2		0.94
Liver/Spleen HU ratio (median, range)	1.3 (1–2)		1.2 (0.1–2)		0.38
Menopausal status					
Pre	53	(42)	21	(46)	0.67
Post	73	(58)	25	(54)	
T					
2	84	(67)	36	(78)	0.33
3	19	(15)	5	(11)	
4	23	(18)	5	(11)	
Histological type					
Ductal	112	(89)	28	(61)	<0.05
Other	14	(11)	18	(39)	
Subtype					
ER (+), HER2 (−)	68	(54)	9	(20)	<0.05
ER (+), HER2 (+)	21	(17)	9	(20)	
HER2	17	(13)	17	(37)	
Triple‐negative	20	(16)	11	(24)	

pCR, pathological complete response; WHO, world health organization; BMI, body mass index; SD, standard deviation; V/S ratio, visceral fat area/subcutaneous fat area ratio; HU, hounsfield unit: ER, estrogen receptor; HER2, human epidermal growth factor receptor 2.

### Adverse events and relative dose intensity

Twenty‐eight patients experienced grade 3 hematological adverse events, of which neutropenia was the most common. The normal weight group had the highest rate of grade 3 adverse events, at 17.1% (19/111). However, in the univariate analysis, BMI was not associated with the occurrence of adverse events (*P* < 0.05; Table 4). The RDI did not differ significantly between BMI groups and was over 98% in all groups (*P* = 0.19; Table 4).

**Table 4:** Relative dose intensity and adverse events during neoadjuvant chemotherapy

	RDI, % (mean ± SD)	*P* a	Hematological adverse events (Grade 3 or 4), *N*	*P* b
WHO BMI classification, kg/m^2^				
Underweight (BMI < 18.5; *n *=* *18)	100 ± 0.0	0.19	2	0.09
Normal range (18.5 ≤ BMI < 25; *n *=* *111)	98.9 ± 3.8		19	
Overweight (25 ≤ BMI <30; *n *=* *35)	99.9 ± 0.2		4	
Obesity (BMI ≥ 30; *n *=* *8)	98.6 ± 1.9		3	

RDI, relative dose intensity; SD, standard deviation; WHO, World Health Organization; BMI, body mass index.

a One‐way analysis of variance.

b Chi square test.

### Body fat distribution and survival

**Figure 1:**
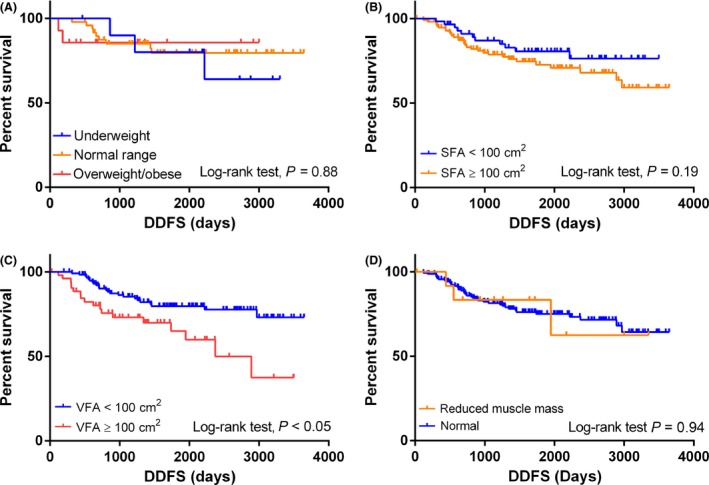
Distant disease‐free survival (DDFS) stratified by body mass index (BMI) and body fat distribution (BFD) parameters. (A) DDFS stratified by BMI. (B) DDFS stratified by subcutaneous fat area (SFA). (C) DDFS stratified by visceral fat area (VFA). (D) DDFS stratified by L3 skeletal muscle index.

The median follow‐up time was 1638 days. DDFS curves stratified by BMI and BFD parameters are shown in Figure 1A–D. The DDFS did not differ significantly according to the BMI group. Furthermore, no significant difference in DDFS was observed when the overweight and obese groups were combined (log‐rank test; *P* = 0.88).

The DDFS was significantly worse for the high VFA group (VFA ≥ 100 cm^2^) compared with the low VFA group (VFA < 100 cm^2^; log‐rank test; *P* < 0.05, HR: 2.36, 95% CI: 1.27−4.38). In contrast, the DDFS did not differ significantly according to the SFA, LSMI, or L/S (data not shown).

**Figure 2:**
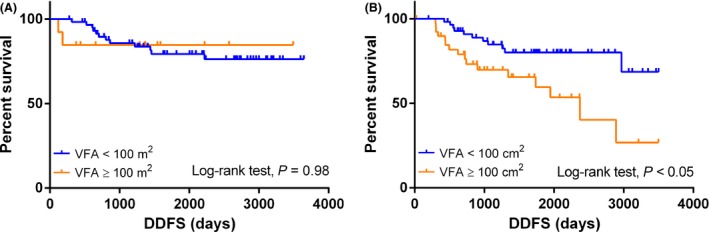
Distant disease‐free survival (DDFS) in the two visceral fat area (VFA) groups stratified by menopausal status. (A) DDFS in premenopausal patients. (B) DDFS in postmenopausal patients.

We further performed an exploratory analysis to determine the impact of menopausal status on the relationship between VFA and DDFS. DDFS did not differ significantly between patients with low or high VFA in the premenopausal group (log‐rank test: *P* = 0.082; Fig. 2A). However, in the postmenopausal group, the DDFS was significantly worse in patients with a high VFA compared to those with a low VFA (log‐rank test: *P* < 0.05, HR: 2.79, 95% CI: 1.29−6.05; Fig. 2B). Furthermore, multivariate analysis revealed that VFA was an independent prognostic factor for DDFS (*P* < 0.05, HR: 2.42, 95% CI: 1.28−4.57; Table 5). The subtype and pCR were also independent prognostic factors for DDFS. Overall survival analysis according to BMI and BFD parameters was also performed, although no significant differences were observed (data not shown).

**Table 5:** Cox proportional hazards regression analysis of distant disease‐free survival

Variables	HR	95% CI	*P*
pCR			
No	1.00		<0.05
Yes	0.21	0.08–0.56	
Subtype			
ER (+), HER (−)	1.00		<0.05
ER (+), HER (+)	1.88	0.70–5.11	
HER2	5.62	2.49–12.68	
Triple‐negative	3.83	1.62–9.03	
VFA			
<100 cm^2^	1.00		<0.05
≥100 cm^2^	2.42	1.28–4.57	
T			
2	1.00		0.47
3	0.92	0.35–2.42	
4	1.54	0.74–3.21	

HR: hazard ratio; CI, confidence interval; pCR, pathological complete response; ER, estrogen receptor; HER2, human epidermal growth factor 2; VFA, visceral fat area.

## Discussion

Despite the numerous studies regarding body composition and treatment outcomes, only a few studies have analyzed the association between body composition and NAC outcomes 8, 24. Our study has two important findings. First, the dominant change in BFD after menopause is increased visceral fat accumulation. This increase occurs regardless of BMI. Second, visceral fat accumulation was strongly associated with survival after NAC, especially in postmenopausal breast cancer patients.

In this study, we found that menopause causes characteristic changes in BFD, including increased VFA and decreased skeletal muscle mass, and these changes are not reflected in the BMI value. The menopause‐related changes in body composition in our study are consistent with those reported in previous studies 25, 26. However, the lack of effect of menopause on liver fat accumulation was unexpected, given the increase in the other BFD parameters. Only 18 patients in our study had fatty liver, suggesting that the incidence of nonalcoholic fatty liver disease in obese patients may be lower than expected. In a systematic review, the mean morbidity rate for nonalcoholic fatty liver disease among obese patients was reported to be 33% 5. Confounding factors make it difficult to determine whether fatty liver or visceral fat worsen prognosis. However, our findings suggest that visceral fat accumulation has a significant negative effect on breast cancer prognosis.

Recently, the relationship between sarcopenic obesity and cancer mortality has become a major area of interest in the cancer nutrition field. Sarcopenic obesity, defined as a combination of excess weight and reduced muscle mass, leads to poor prognosis by lowering functional status and increasing chemotherapy‐related morbidity and toxicity 12. Only a few patients had sarcopenia or sarcopenic obesity in this study, and muscle depletion had no significant effect on survival outcome. The result may came from the fact that present threshold value for LSMI was higher than previous studies, ranging from 38.5 to 41.0 cm^2^/m^2^ according to the optimal stratification, hence the distribution may be inappropriate for evaluating the effect of sarcopenia on survival 12, 27, 28. However, patients with presarcopenia may have a higher risk of worsening unfavorable body composition changes after chemotherapy or endocrine therapy. Although we only evaluated body composition at the time of the initial treatment in this study, changes in body composition also need to be monitored during the follow‐up period to prevent sarcopenic obesity.

In this study, we demonstrated that patients with a high amount of visceral fat had a significantly shorter DDFS, even though VFA was not associated with chemosensitivity (i.e., the pCR rate). When determining the effect of obesity on NAC outcomes, both the chemosensitivity to NAC (pCR rate) and survival after NAC must be considered. The largest study (*n* = 1169) investigating the relationship between NAC chemosensitivity and obesity in breast cancer was conducted at the MD Anderson Cancer Center in 2008. In this study, the pCR rate was significantly lower in overweight/obese patients (BMI ≥ 25 kg/m^2^) than in normal/underweight patients (BMI < 25 kg/m^2^) 1. In contrast, a meta‐analysis of eight major clinical trials found no significant association between obesity and chemosensitivity to NAC 29. Thus, the impact of obesity on NAC chemosensitivity remains controversial. On the other hand, studies have consistently shown that obese patients have a significantly worse survival outcome after NAC 1, 3, 29, 30. Together, these studies indicate that obesity has a greater impact on survival outcomes after NAC than NAC chemosensitivity. Obesity promotes cancer progression by increasing cell proliferation, cell survival, invasion/metastasis, and angiogenesis. These effects are mediated by the induction of insulin resistance and inflammation, and increases in leptin, adiponectin, plasminogen activator inhibitor‐1, and vascular endothelial growth factor levels 6. In particular, visceral fat, which is more hormonally active than other types of body fat, strongly promotes these effects 6, 31. Thus, the shorter DDFS in the high visceral fat group in our study may be due to the positive effect of visceral fat on cancer progression.

## Conclusion

To date, much attention has been paid to increasing the pCR rate after NAC, whereas little emphasis has been placed on supportive care during the NAC follow‐up period. However, a recent study demonstrated the importance of supportive care, including nutritional support and physical exercise, for improving breast cancer survival 32. Thus, a multidisciplinary approach including nutritional support and physical exercise is needed to improve survival outcomes after NAC in obese breast cancer patients.

## Conflict of Interest

None declared.
